# Macromolecular Model Construction and Pore Structure Distribution of Coals with Different Ranks

**DOI:** 10.3390/molecules31081289

**Published:** 2026-04-15

**Authors:** Xiaoyue Zhao, Xihua Zhou, Yu Cao

**Affiliations:** 1 College of Safety Science & Engineering, Liaoning Technical University, Huludao 125105, China; 2 Key Laboratory Mine Thermodynam Disasters & Control, Ministry of Education, Liaoning Technical University, Huludao 125105, China

**Keywords:** molecular structural characteristics, pore development in coal, low-temperature N_2_ adsorption, oxygen-containing functional groups, occurrence modes of elements

## Abstract

This study investigates lignite, long-flame coal, coking coal, and anthracite to elucidate the rank-dependent evolution of coal macromolecular structure and pore systems. Elemental/proximate analyses, FTIR, XPS, ^13^C NMR, and low-temperature N_2_ adsorption–desorption, combined with BET, BJH, and DFT models, were employed to quantify structural parameters, characterize pore-size distributions, and establish representative macromolecular models. The results show that coalification is accompanied by progressive aromatization and polycondensation. Low-rank coal contains abundant hydroxyl, carboxyl, and aliphatic side-chain structures, exhibiting low aromaticity and weak aromatic-ring condensation. With increasing rank, oxygen-containing groups and aliphatic chains are progressively removed, while aromatic carbon content and the bridgehead-to-peripheral carbon ratio increase markedly. High-rank coal is dominated by highly condensed aromatic lamellae, with lower molecular polarity and enhanced structural ordering and graphitization. Meanwhile, N and S occurrence modes evolve from edge-related reactive species to more stable heterocyclic configurations, reflected by increasing graphitic N and thiophenic S contents. Pore structures also change systematically: low-rank coal is characterized by open, multimodal mesopores; intermediate-rank coal shows compaction and mesopore collapse; and high-rank coal becomes micropore-dominated with a relatively closed network. The U-shaped variation in micropore and mesopore volumes with rank indicates coupled macromolecular polycondensation and pore reconstruction, providing a structural basis for spontaneous combustion propensity and coalbed methane occurrence.

## 1. Introduction

Coal is a key fossil energy and strategic resource. Its physicochemical properties directly control its behavior in energy conversion, coalbed methane exploitation, underground gasification, and coal chemical utilization. From a structural perspective, coal is a complex, three-dimensional cross-linked macromolecular assemblage composed of aromatic structural units, aliphatic bridges, and a variety of oxygen-, nitrogen-, and sulfur-containing functional groups. The structure is highly heterogeneous and exhibits a strong dependence on coal rank [1]. With ongoing coalification, coal evolves from low to high rank, accompanied by systematic changes in macromolecular structure, heteroatom occurrence, and the pore network. Therefore, elucidating coal evolution simultaneously from molecular and pore-structure perspectives is essential for understanding reactivity, gas adsorption, and thermal stability.

Traditionally, studies on coal macromolecular structure have focused on parameters such as aromaticity, the aliphatic carbon fraction, and the bridgehead carbon ratio. FTIR provides effective constraints on hydroxyl, carboxyl, aliphatic, and aromatic structural features; solid-state ^13^C NMR enables quantitative discrimination between aliphatic and aromatic carbon, thereby offering a direct basis for constructing macromolecular structural models; XPS is particularly useful for resolving the occurrence states of heteroatoms such as N and S. In recent years, multi-technique integration has become a mainstream approach for probing coal microstructural evolution. Meanwhile, coal is a typical multiscale porous medium whose pore system controls adsorption, diffusion, and storage of gases. Low-temperature N_2_ adsorption–desorption combined with BET, BJH, and DFT models allows systematic characterization of specific surface area, pore-size distribution, and pore volume. Existing evidence suggests a close coupling between molecular and pore structures: polycondensation of aromatic domains and compaction of the carbon skeleton can trigger pore collapse and reconstruction, whereas removal of oxygen-containing groups alters pore-wall polarity and adsorption affinity.

Since the 1980s, extensive work has been devoted to average structural model construction, three-dimensional reconstruction, and molecular dynamics simulation of coal. Representative early studies include structural bases for coal pyrolysis proposed by Solomon [2] and co-workers and the statistical structural model concept developed by Given [3]. In the 1990s, Wiser established a more systematic framework for average structural models based on elemental analysis, solid-state ^13^C NMR, and infrared data [1]. Faulon introduced computer-assisted stochastic assembly to generate three-dimensional coal models constrained by experimental parameters, which is widely regarded as a key milestone in digital coal modeling [4]. More recently, Mathews et al. developed a refined three-dimensional model for Illinois No. 6 bituminous coal and verified its density and pore-related parameters via molecular dynamics, highlighting a shift from two-dimensional average structures to three-dimensional statistical representations [5]. Ungerer and co-workers further applied molecular simulation to coal–CO_2_/CH_4_ interactions, promoting the use of coal molecular models in CBM and CO_2_ geological storage. In China, researchers such as Huang XH and Zhang SY constructed average macromolecular models for coals of different ranks using combined ^13^C NMR, XPS, and FTIR constraints [6].

Systematic research on coal pore-size distributions has also progressed rapidly. Qiao, YG et al. used low-temperature gas adsorption and CO_2_ adsorption to demonstrate that micropores dominate adsorption capacity [7]. Clarkson combined mercury intrusion and gas adsorption to show that pore-size distributions vary markedly with rank: low-rank coals tend to contain more meso-/macropores, whereas microporosity becomes more prominent at higher rank [8]. In recent years, small-angle X-ray scattering (SAXS) and FIB-SEM-based three-dimensional reconstruction have enabled quantitative characterization of nanoscale pores. In China, Liu Honglin and co-workers analyzed fractal features of coal-reservoir pore structures [9], and Ranran Hou investigated the in situ observation and quantitative analysis of lignite, as well as the effect of hydrogen bond-inhibited cross-linking reactions on thermal conversion [10].

Against this background, four coal samples of different ranks were selected in this work. Elemental analysis, FTIR, XPS, ^13^C NMR, and low-temperature N_2_ adsorption–desorption were employed to systematically investigate macromolecular structural parameters, heteroatom occurrence, and pore development. Representative macromolecular models were then constructed for different ranks, and the coupling between molecular evolution and pore reconstruction was examined. Through a multiscale, multi-parameter assessment, this study aims to identify structural essentials along the coalification pathway and to clarify how such changes shape the pore system, thereby providing theoretical support for efficient utilization and safe extraction of coal resources. Coalification alters the chemical structural characteristics, total pore volume, specific surface area, pore-size distribution, and pore complexity of coal; in turn, these changes impact the adsorption, diffusion, and percolation of methane. Moreover, this influence is often nonlinear. Since the pore system dictates both the mode of coalbed methane occurrence and the efficiency of extraction, a multiscale evaluation approach facilitates the optimal selection of extraction intervals and operational sequences. This enables the transformation of gas—which would otherwise constitute a safety hazard—into a usable energy resource, thereby reducing the time required for extraction and preparatory work, mitigating accidents such as coal outbursts and spontaneous combustion, and ultimately enhancing overall resource recovery rates while simultaneously ensuring safety.

## 2. Results

### 2.1. Basic Coal Quality Analysis

Vitrinite reflectance was measured according to GB/T 6948–2008 [11]. Proximate analysis was conducted following GB/T 212–2008 [12]. Elemental and proximate analysis results of coal samples are summarized in Table 1.

### 2.2. FTIR-Based Analysis of Functional Groups

#### 2.2.1. Aromatic Region (900–700 cm^−1^)

**Figure 1:**
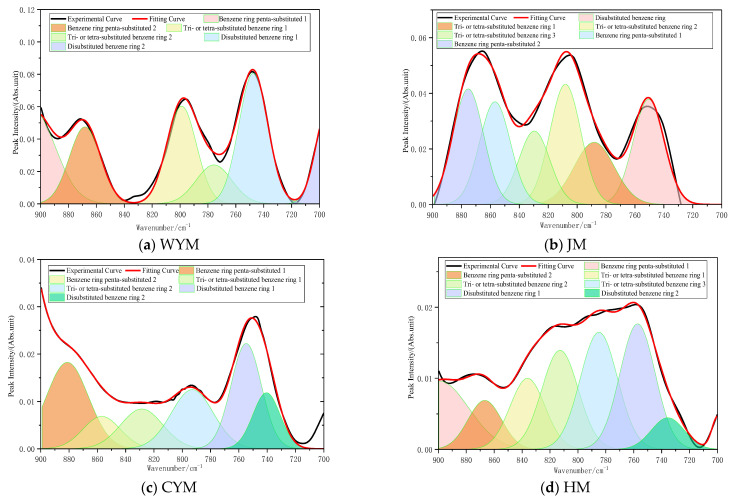
Assignment of functional groups in the aromatic region (900–700 cm^−1^).

The 900–700 cm^−1^ region is dominated by out-of-plane C–H bending vibrations of aromatic hydrocarbons and is commonly used to infer aromatic-ring substitution patterns. In lignite (HM), aromatic structures are relatively underdeveloped and substitution forms are diverse [13]. Aliphatic moieties and oxygen-containing groups dominate the macromolecular framework; consequently, the overall absorption intensity in this region is weak, and the bands tend to be dispersed and overlap as multiple peaks [14]. Such features suggest a heterogeneous distribution of substituted aromatic rings and a low extent of aromatic condensation, consistent with structural instability, high polarity, and abundant reactive sites [15]. The assignment of functional groups in the aromatic region is shown in Figure 1.

For the long-flame coal (CYM), ortho-disubstituted and para-substituted peaks become more discernible, implying that the number of substituents around certain aromatic rings decreases and the structure becomes comparatively more regular. Following cleavage of aliphatic side chains, the degree of aromatic substitution declines and the fraction of mono-substituted rings increases. These changes indicate a transition from highly random substitution toward a more concentrated substitution mode, accompanied by gradual growth of aromatic lamellae—an important intermediate stage from low- to medium-rank coal.

During coalification to the coking coal stage (JM), extensive removal of side chains and oxygenated substituents reduces the number of substituents around aromatic rings and drives substitution patterns toward simplicity. As a result, ortho- and meta-substitution bands weaken, whereas mono- or sparsely substituted ring signatures become dominant. This behavior is consistent with condensation between aromatic structures to form larger conjugated systems, thereby substantially increasing skeleton stability. In this rank range, aromatic structures become the core determinant of physicochemical properties.

In anthracite (WYM), most aliphatic and oxygen-containing substituents have been removed, leaving aromatic rings largely exposed or connected only by a small number of bridging motifs. The band intensity in this region remains stable but does not increase markedly, reflecting enhanced ordering and a tendency toward graphitization. Intermolecular interactions are increasingly governed by π–π stacking, while polar interactions give way to van der Waals forces and conjugation-related interactions, yielding the highest structural stability among the samples.

#### 2.2.2. Oxygen-Containing Functional Groups (1800–1000 cm^−1^)

**Figure 2:**
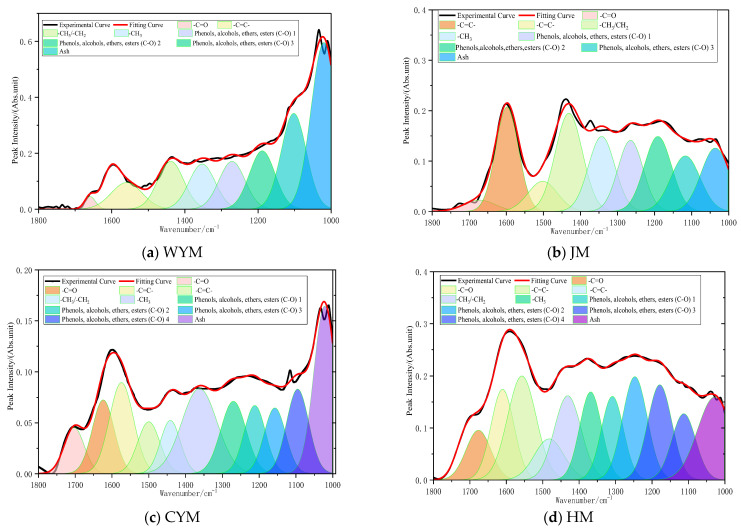
Assignment of oxygen-containing functional groups (1800–1000 cm^−1^).

The 1800–1000 cm^−1^ interval contains the richest structural information in coal FTIR spectra, encompassing a variety of oxygen-containing functional groups as well as certain aliphatic deformation modes [16]. Absorption intensity in this region is strongly correlated with oxygen functionality abundance. As a low-rank coal, lignite (HM) preserves substantial residual biogenic structures and is therefore enriched in oxygenated groups. A pronounced C=O stretching band near 1700 cm^−1^ is typically observed, attributable to carboxylic acids, ketones, aldehydes, and esters. Strong C–O stretching in the 1250–1050 cm^−1^ region indicates abundant alcohol, phenolic, and ether structures. In contrast, the aromatic C=C band around 1600 cm^−1^ is relatively weak, reflecting limited aromatic condensation. Collectively, low-rank coal exhibits the characteristic pattern of ‘strong oxygenated bands and weak aromatic bands’, implying high polarity and hydrogen-bond/dipole-dominated intermolecular forces. Such structural features contribute to high reactivity during low-temperature oxidation and a stronger propensity for spontaneous combustion [17]. The assignment of oxygen-containing functional groups is shown in Figure 2.

With increasing rank, decarboxylation, dehydration, and condensation reactions proceed. In the long-flame coal (CYM), the C=O band at ~1700 cm^−1^ decreases, indicating depletion of carboxylic structures; the C–O band in the 1200–1000 cm^−1^ range also weakens as alcohol/ether structures are progressively cleaved. Meanwhile, the aromatic C=C band near 1600 cm^−1^ strengthens, indicating a higher proportion of aromatic structures. In this stage, oxygenated and aromatic bands may be comparable or alternate in dominance, suggesting a critical transition from an oxygen-dominated to an aromatic-dominated framework. Mechanistically, this corresponds to accelerated aliphatic cracking and aromatization, which enhances stability while preserving some reactive sites.

At the coking coal stage (JM), the C=O band diminishes further, leaving only small amounts of relatively stable carbonyl species; the C–O band continues to decline, indicating substantial oxygen loss. The aromatic C=C band becomes dominant in this region, demonstrating that aromatic rings have become the principal skeleton component. In anthracite (WYM), oxygen-containing groups reach a minimum: C=O and C–O bands are nearly absent, whereas the aromatic skeleton band at ~1600 cm^−1^ is most prominent. Overall, this spectral region follows a clear coalification trend of decreasing oxygenated-band intensity and increasing aromatic-band intensity.

#### 2.2.3. Aliphatic C–H Stretching Region (3000–2800 cm^−1^)

**Figure 3:**
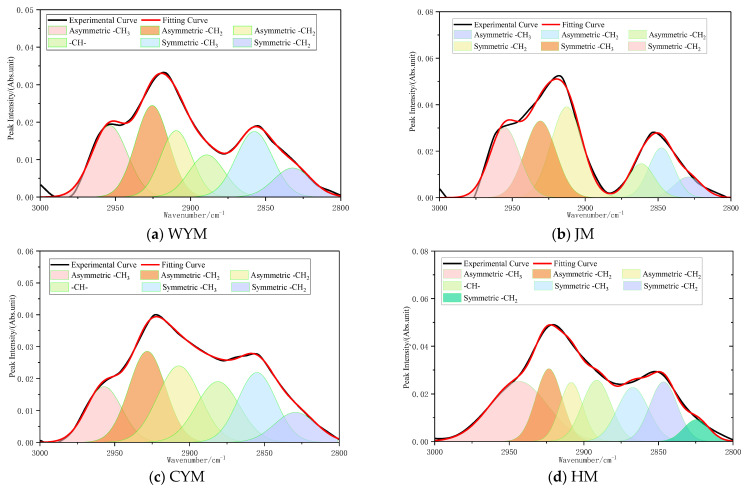
Assignment of functional groups in the aliphatic C–H stretching region (3000–2800 cm^−1^).

The 3000–2800 cm^−1^ region corresponds mainly to stretching vibrations of aliphatic –CH_3_ and –CH_2_– groups and is therefore informative for side-chain development. Low-rank coals commonly contain abundant aliphatic side chains, with coexisting long-chain alkyl and branched structures. Accordingly, strong bands at ~2920 cm^−1^ (asymmetric –CH_2_–), ~2850 cm^−1^ (symmetric –CH_2_–), and ~2950 cm^−1^ (asymmetric –CH_3_) are typically observed. A relatively high CH_2_/CH_3_ area ratio suggests the dominance of long-chain aliphatic structures, consistent with the preservation of biogenic remnants. The assignment of functional groups in the aliphatic C–H stretching region is shown in Figure 3.

With increasing rank, aliphatic structures are progressively depleted: the –CH_2_– band weakens markedly, and only limited bridging or short side-chain structures remain. The –CH_3_ band also decreases, although its relative share may become slightly higher than that of –CH_2_–, consistent with methyl substitution on aromatic rings. Overall, the absorption intensity in this region becomes far lower than that of low-rank coal, indicating that the carbon skeleton is increasingly aromatic, planar, and highly condensed, with aliphatic side chains approaching extinction in high-rank coal.

#### 2.2.4. Hydroxyl Band (3600–3000 cm^−1^)

**Figure 4:**
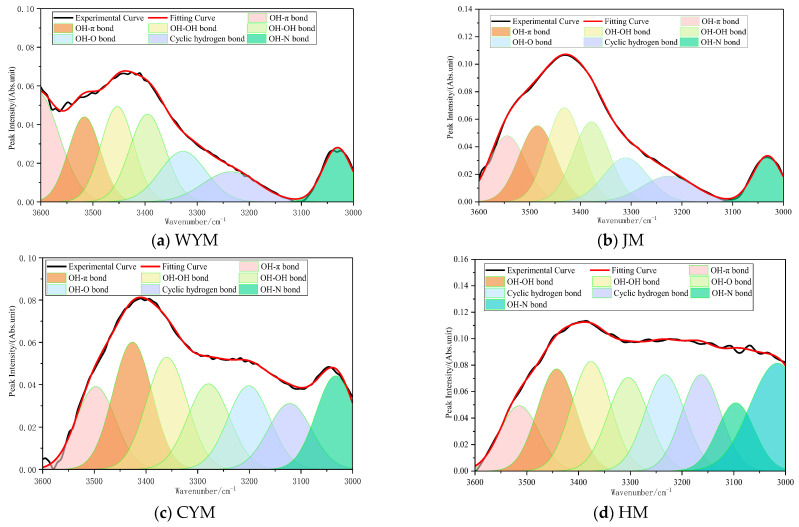
Assignment of functional groups in the hydroxyl band (3600–3000 cm^−1^).

The hydroxyl band (3600–3000 cm^−1^) arises mainly from phenolic hydroxyls (Ar–OH), alcohol hydroxyls, and –OH in carboxyl groups. Because –OH stretching is highly sensitive to rank, it serves as an indicator of polar structures and hydrogen bonding networks. In lignite (HM), oxygenated groups are abundant, particularly phenolic, alcoholic, and carboxylic moieties. Strong intermolecular and intramolecular hydrogen bonding yields a broad, intense envelope near ~3400 cm^−1^. Band broadening reflects multiple coexisting hydrogen-bond configurations, including OH–OH, OH–π, and OH–O=C interactions, consistent with a loose and highly polar structure. The assignment of functional groups in the hydroxyl band is shown in Figure 4.

As coalification advances to the long-flame coal stage (CYM), decarboxylation, dehydration, and condensation remove unstable oxygenated structures, reducing hydroxyl abundance and weakening the hydrogen-bond network. The band intensity decreases; the envelope becomes narrower; and the peak position may shift slightly to higher wavenumbers, indicating a reduced fraction of strongly hydrogen-bonded hydroxyls and a higher proportion of free or weakly associated hydroxyls. At the coking coal stage (JM), continued aromatization and condensation further reduce hydroxyl groups, leaving only minor phenolic or mineral-related contributions. In anthracite (WYM), oxygenated groups are largely exhausted; only very weak absorption remains, often attributable to adsorbed water or trace residual hydroxyls. Intermolecular interactions become dominated by π–π stacking. Thus, hydroxyl band intensity decreases monotonically with increasing rank, reflecting the ‘deoxygenation–aromatization–stabilization’ pathway.

#### 2.2.5. FTIR-Derived Structural Parameters

Aromatic carbon ratio, degree of condensation, and aliphatic chain length are basic parameters for coal macromolecular-model construction. Following the approach in Refs., peak-area integration was employed to quantify key structural indices.

(1) Aliphatic chain length. The aliphatic chain length was estimated using the –CH_3_/–CH_2_ peak-area ratio. A smaller value indicates a longer aliphatic chain. The calculated values were 1.18 for WYM, 1.25 for JM, 1.67 for CYM, and 1.80 for HM.

(1) $$\frac{A_{1}\left(CH_{2}\right)}{A_{1}\left(CH_{3}\right)}=\frac{A_{1}\left(2900\sim 2940\;cm^{-1}\right)}{A_{1}\left(2940\sim 3000\;cm^{-1}\right)}$$

(2) Aromaticity describes the richness of aromatic relative to aliphatic functionalities. The calculated aromaticity values were 2.47 for WYM, 1.55 for JM, 0.68 for CYM, and 0.57 for HM.

(2) $$I=\frac{A_{1}\left(900\sim 700\;cm^{-1}\right)}{A_{1}\left(3000\sim 2800\;cm^{-1}\right)}$$

(3) Degree of aromatic-ring condensation (DOC). DOC was defined as the ratio between the aromatic C–H out-of-plane deformation in the 900–700 cm^−1^ region and the aromatic C=C skeletal vibration near 1600 cm^−1^. The DOC values were 0.64 for WYM, 0.26 for JM, 0.12 for CYM, and 0.11 for HM.

(3) $$DOC=\frac{A_{1}\left(900\sim 700\;cm^{-1}\right)}{A_{1}\left(1600\;cm^{-1}\right)}$$

### 2.3. Occurrence of N and S in Coal

#### 2.3.1. Nitrogen Speciation

**Figure 5:**
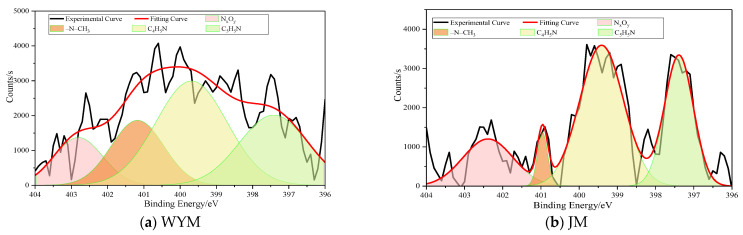

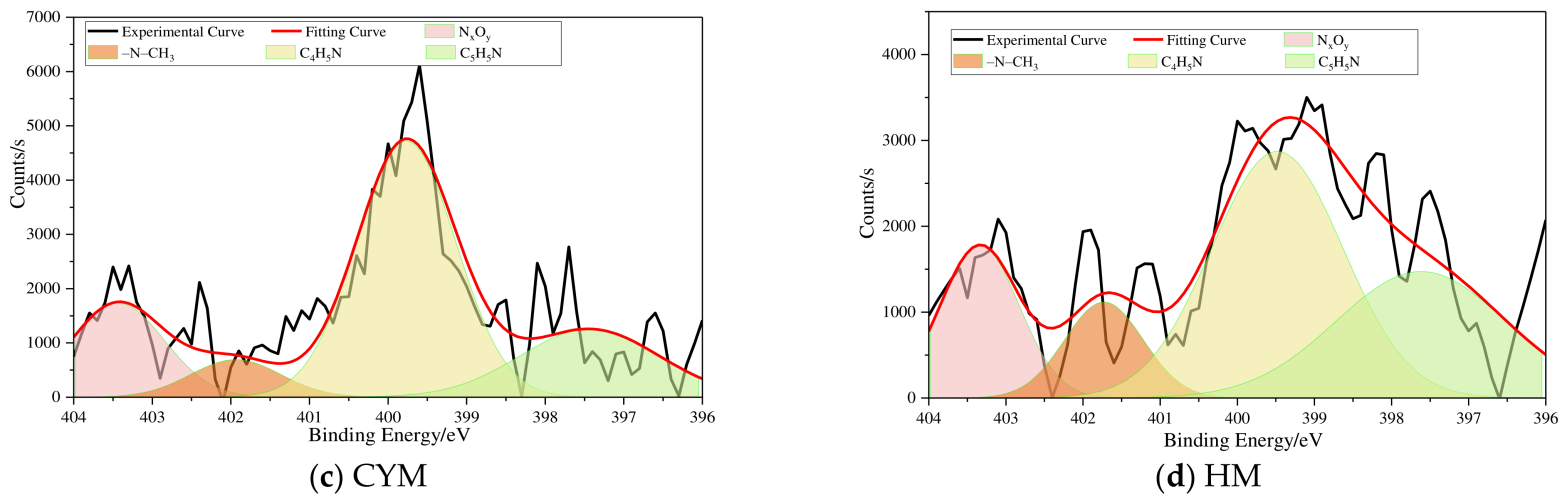
Occurrence of N in coal.

Peak deconvolution indicates that nitrogen in coal mainly occurs as pyridinic N (N-6), pyrrolic N (N-5), quaternary/graphitic N (N-Q), and oxidized N (N-X). Pronounced rank-dependent differentiation is observed [1,18]. In lignite (HM), the relative proportions of N-5 and N-X are comparatively high, suggesting that nitrogen largely resides at reactive edges of heteroaromatic structures or in oxidized configurations (e.g., amide- or nitro-like species). Given the low degree of polycondensation, nitrogen is commonly incorporated into five-membered rings or exists in less stable oxygenated nitrogen functionalities, leading to higher structural reactivity. The occurrence of N in coal is shown in Figure 5.

At the long-flame coal stage (CYM), the fraction of N-5 decreases while N-6 increases, indicating a transformation from five-membered ring nitrogen toward more stable six-membered aromatic heterocycles. Strengthened deoxygenation and condensation reduce unstable oxidized nitrogen, and nitrogen becomes increasingly embedded in the aromatic framework.

In coking coal (JM) and especially in anthracite (WYM), both N-6 and N-Q increase further; the rise in N-Q (graphitic nitrogen) is particularly evident. This suggests that nitrogen progressively becomes incorporated within aromatic lamellae and participates in π-conjugated systems, yielding higher stability and reflecting substantial aromatization during coalification.

#### 2.3.2. Sulfur Speciation

**Figure 6:**
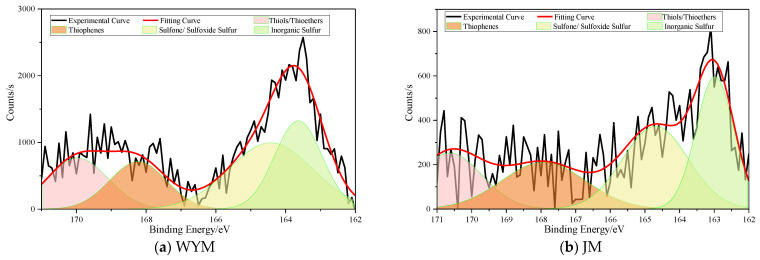

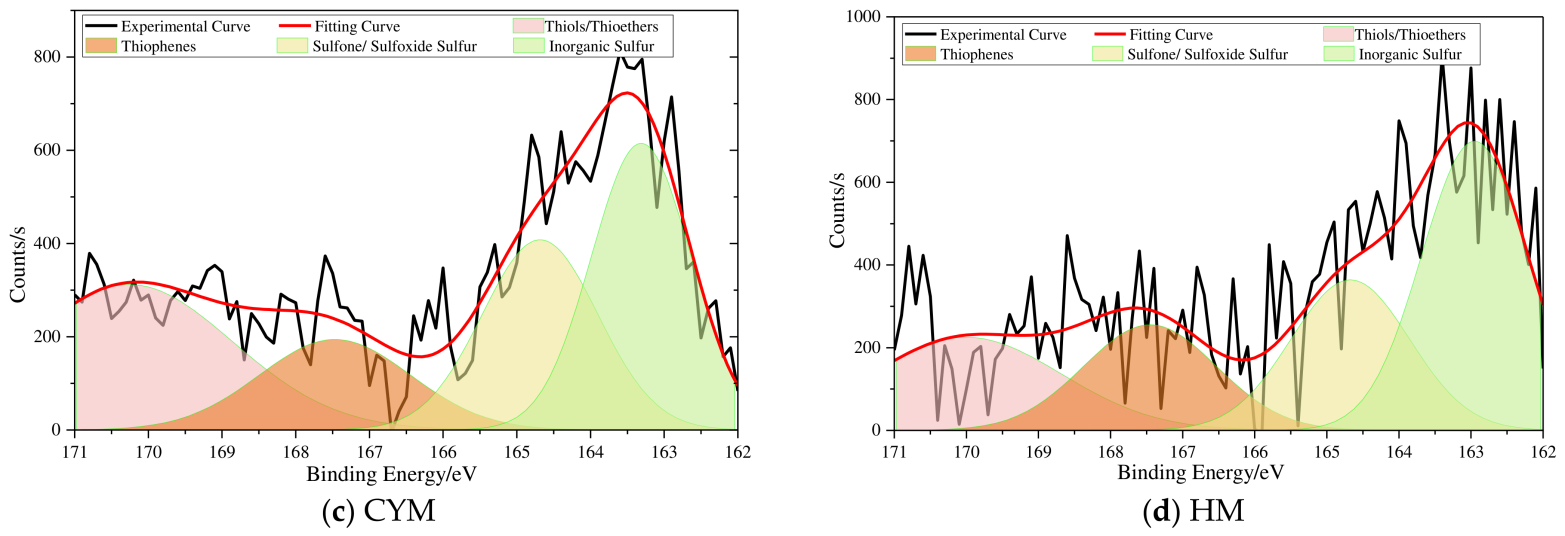
Occurrence of S in coal.

Sulfur in coal comprises organic sulfur (thiophenic S, thioether/thiol S, sulfoxide/sulfone S) and inorganic sulfur (pyritic S, sulfate S). In lignite, organic sulfur is relatively abundant, with noticeable contributions from thioether and thiol structures as well as a certain fraction of oxidized sulfur (sulfoxides/sulfones). This indicates that sulfur in low-rank coal is structurally more reactive and is frequently located on aliphatic chains or at aromatic edges, leading to lower stability. Some inorganic sulfur (e.g., pyrite) may also be present but is not strongly bound to the organic matrix. The occurrence of S in coal is shown in Figure 6.

With coalification to the long-flame coal stage, thioether/thiol fractions decrease, whereas thiophenic sulfur begins to increase. As thiophenic S represents a stable sulfur configuration embedded within aromatic rings, its growth suggests that sulfur becomes involved in aromatization and condensation. Oxidized sulfur decreases correspondingly, indicating stabilization. At the coking coal stage, thiophenic S becomes the dominant organic sulfur form, clearly exceeding low-rank levels. Sulfur is more frequently incorporated into aromatic lamellae as stable heterocycles. In anthracite (WYM), thiophenic sulfur reaches the highest proportion and becomes the overwhelmingly dominant sulfur form; inorganic sulfur is relatively low and occurs mainly as stable pyrite. Oxidizable sulfur is scarce, indicating substantial sulfur stabilization and incorporation into the conjugated aromatic system at high rank.

### 2.4. Carbon Skeleton Characteristics

Considering the coalification pathway and the structural features inferred from the spectra, the occurrence of the carbon skeleton across coal ranks can be discussed in terms of the size of aromatic structural units, condensation degree, retention of side chains, and spatial stacking of carbon frameworks [19,20].

**Figure 7:**
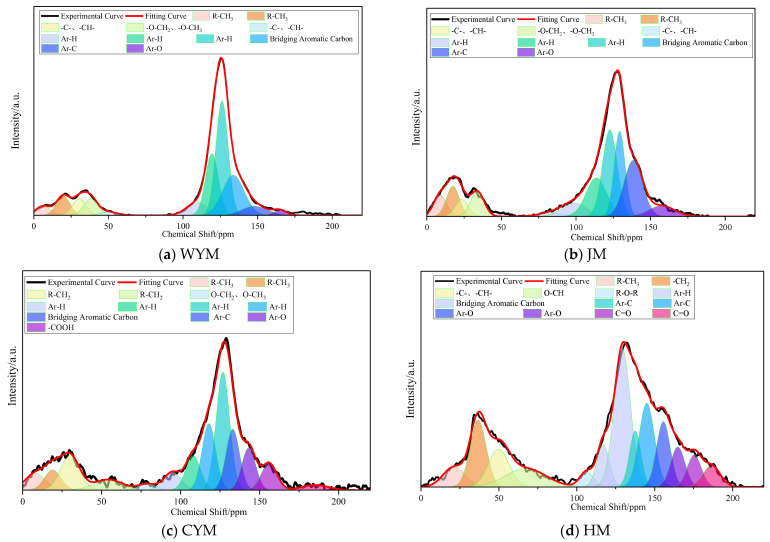
Peak fitting spectra of ^13^C NMR for different coal samples.

Comparing the four coals reveals a clear evolutionary pattern from HM to WYM: with increasing rank, aromaticity increases and aromatic cores expand; aliphatic side chains and oxygen-containing groups are gradually removed; aromatic lamellae evolve from dispersed small units to larger plate-like domains; the arrangement transitions from high disorder toward short-range ordering; and the carbon skeleton changes from a cross-linked network to a lamellar stacking architecture. Fundamentally, this evolution reflects persistent dehydration, decarboxylation, demethylation, aromatization, and condensation reactions during coalification. The peak fitting spectra of ^13^C NMR for different coal samples are shown in Figure 7.

Accordingly, the carbon skeleton exhibits stage-dependent occurrence: HM is characterized by small aromatic cores embedded in a highly cross-linked network; CYM shows medium-sized aromatic lamellae with semi-ordered stacking; JM develops larger aromatic lamellae and a turbostratic microcrystalline character; and WYM is dominated by highly condensed large aromatic lamellae with quasi-graphitized stacking.

The ^13^C NMR spectra can be partitioned into two major clusters: aliphatic carbon (f_al_ = f_al_* + f_al_^H^ + f_al_^O^) and aromatic carbon (f_a_′ = f_a_^H^ + f_a_^N^ = f_a_^H^ + f_a_^P^ + f_a_^S^ + f_a_^B^). A chemical shift of 70 ppm is used as the boundary between these clusters. Structural parameters derived from ^13^C NMR of coal samples are summarized in Table 2.

The bridgehead carbon ratio (X_BP_) can be used to characterize aromatic condensation in the macromolecular structure. Substituting the parameters from Table 3 indicates that X_BP_ increases with coal rank, implying a higher degree of aromatic condensation in higher-rank coals. The X_BP_ values were 0.32 for WYM, 0.27 for JM, 0.20 for CYM, and 0.11 for HM. The aromatic carbon fractions (f_a_) of the four coals were 80.16%, 75.96%, 71.06%, and 70.33%, whereas the aliphatic carbon fractions (f_al_) were 19.78%, 23.98%, 28.86%, and 29.50%, respectively. These results suggest that high-rank coal has a higher aromatic carbon ratio and more readily forms high-ring-number aromatic compounds, while lower-rank coal contains comparatively more low-ring-number aromatics. Across all ranks, aromatic carbon dominates the molecular composition, whereas aliphatic carbon mainly serves as connecting segments between aromatic structural units.

### 2.5. Construction of Coal Macromolecular Models

To determine the molecular formulae of representative coal macromolecules, the atomic ratios H/C, O/C, N/C, and S/C (denoted as A, B, C, and D) were calculated from elemental analysis. A general formula can then be written as C_x_H_Ax_O_Bx_N_Cx_S_Dx_. Numerous studies suggest that the molecular weight of a representative coal macromolecule is approximately 2000–3000. Accordingly, the molecular weight should satisfy:

12x + Ax + 16Bx + 14Cx + 32Dx > 2000     12x + Ax + 16Bx + 14Cx + 32Dx < 3000(4)

where x, Ax, Bx, Cx, and Dx are positive integers. Based on these constraints, the derived molecular formulae were C_203_H_103_O_4_N_5_ for WYM, C_190_H_136_O_8_N_2_ for JM, C_165_H_105_O_21_NS for CYM, and C_164_H_142_O_31_N_2_ for HM.

**Figure 8:**
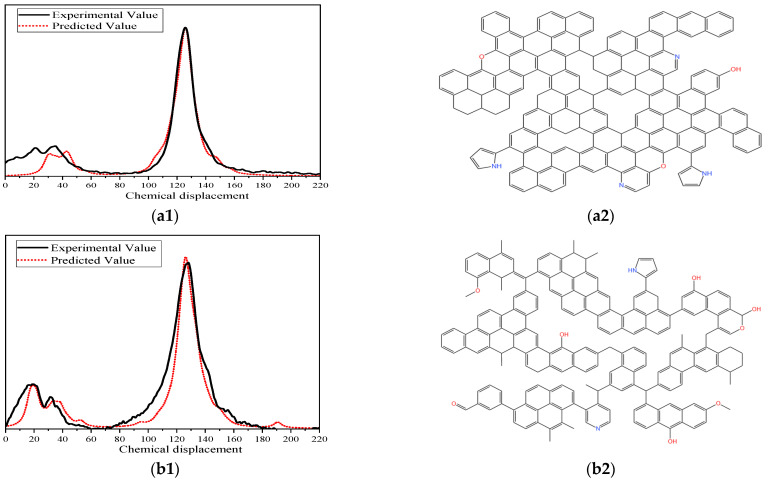

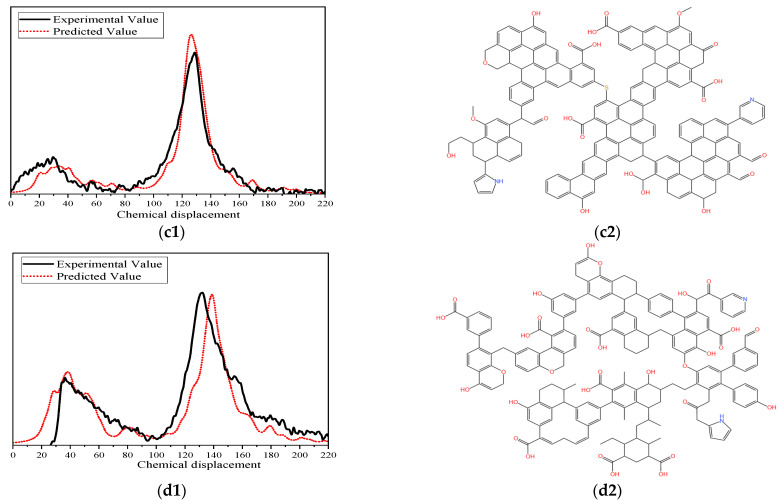
Planar model of the macromolecular structure of coal and its verification diagram. (**a1**) Comparison of WYM experimental spectra and model-calculated spectra. (**a2**) WYM macromolecular structural planar model. (**b1**) Comparison of JM experimental spectra and model-calculated spectra. (**b2**) JM macromolecular structural planar model. (**c1**) Comparison of CYM experimental spectra and model-calculated spectra. (**c2**) CYM macromolecular structural planar model. (**d1**) Comparison of HM experimental spectra and model-calculated spectra. (**d2**) HM macromolecular structural planar model.

The X_BP_ values for benzene, naphthalene, anthracene, phenanthrene, and pyrene units are 0, 0.25, 0.40, and 0.50, respectively, whereas pyrrole, pyridine, and thiophene units have X_BP_ = 0 [21]. Let the numbers of benzene, naphthalene, anthracene/phenanthrene, pyrene, pyrrole, pyridine, and thiophene units be a, b, c, d, e, f, and g, respectively. The counts of different aromatic structural units in coal models are listed in Table 3. Based on the number of different aromatic structural units obtained from the above tests, models of coal with different degrees of metamorphism were drawn. The planar model of the macromolecular structure of coal and its verification diagram are shown in Figure 8.

### 2.6. Pore-Structure Development

**Figure 9:**
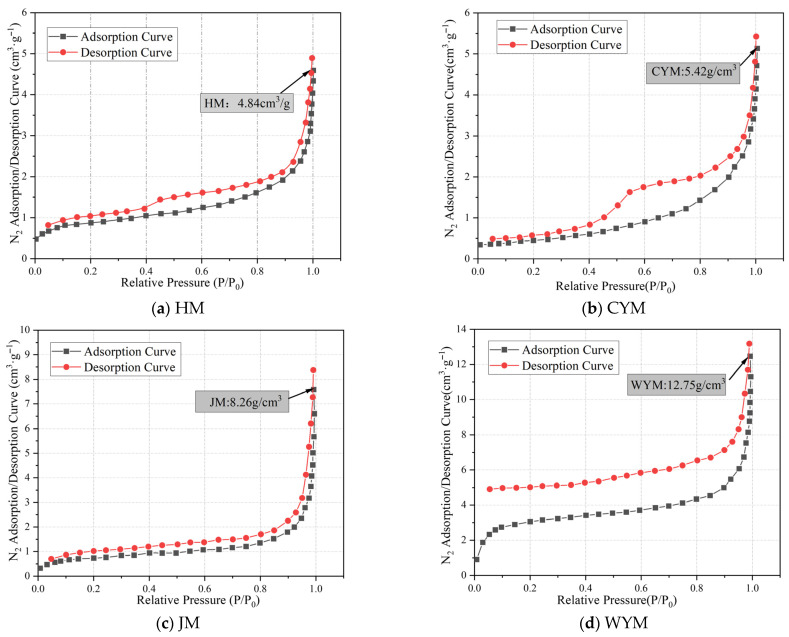
Low-temperature N_2_ adsorption/desorption curves of coal samples.

Low-temperature N_2_ adsorption/desorption curves of coal samples are shown in Figure 9. The adsorption isotherms of different metamorphic degrees are divided into three stages based on the range of relative pressure (P/P_0_), namely the low-pressure stage (0–0.1), the medium-pressure stage (0.1–0.8), and the high-pressure stage (0.8–1).

Based on the presence and width of hysteresis loops in the nitrogen adsorption–desorption isotherms, the pore morphology of different coal samples can be classified into three types: Type I, Type II, and Type III. Low-temperature N_2_ adsorption/desorption curves of coal samples are shown in Figure 9.

Type I mainly corresponds to HM and CYM coal samples. When the relative pressure decreases from 1.0 to 0.5, both exhibit relatively wide hysteresis loops, indicating the development of numerous slit-type mesopores and macropores in the samples, with good pore openness and connectivity. In the relative pressure range of 0.5–0.45, the desorption branch shows a significant decreasing inflection point, indicating the presence of numerous ink bottle-shaped pores in the coal body. When the relative pressure further decreases from 0.45 to 0, the adsorption and desorption branches of CYM basically overlap, while HM still exhibits a certain but relatively narrow hysteresis loop. This phenomenon is a typical low-pressure hysteresis, generally considered to be related to the irreversible adsorption of N_2_ in micropores. Some studies have also suggested that it may originate from adsorption-induced expansion of the pore structure. Based on this, it can be inferred that CYM is dominated by closed micropores, while HM contains a small number of well-connected micropores and a certain number of closed micropores.

Type II mainly corresponds to high-rank coal WYM. Its high-pressure zone characteristics are similar to Type I, but within the relative pressure range of 0.5–0.45, the desorption branch inflection point is not obvious, and a wide hysteresis loop appears in the low-pressure zone, indicating a significant low-pressure hysteresis effect. This suggests that WYM contains only a small number of ink bottle-shaped pores, while a large number of well-connected slit-type mesopores, macropores, and open micropores are more developed.

Type III mainly corresponds to coal sample JM. Its adsorption and desorption branches basically overlap throughout the relative pressure range, and the hysteresis loop is extremely narrow, indicating that this coal sample is dominated by closed micropores and a large number of one-end closed plate-shaped mesopores and macropores, while open slit-type pores are relatively few.

Based on the N_2_ adsorption–desorption results and the BET/BJH/DFT models, the BJH total pore volumes of the four coals range from 0.6845 × 10^−2^ to 1.4562 × 10^−2^ cm^3^·g^−1^, following HM > WYM > CYM > JM. The micropore and mesopore BJH pore volumes range from 0.0012 × 10^−2^ to 0.0359 × 10^−2^ cm^3^·g^−1^ and from 0.2946 × 10^−2^ to 0.9625 × 10^−2^ cm^3^·g^−1^, respectively; both exhibit a U-shaped trend with rank. Mesopores contribute the largest fraction (57.84–70.71%), and low-rank coal generally has a higher mesopore volume fraction than high-rank coal. Micropores contribute the smallest fraction; WYM shows the highest micropore fraction (5.46%) among the samples as reported in the original manuscript.

Pore volume parameters of coal samples derived from N_2_ adsorption are listed in Table 4.

**Figure 10:**
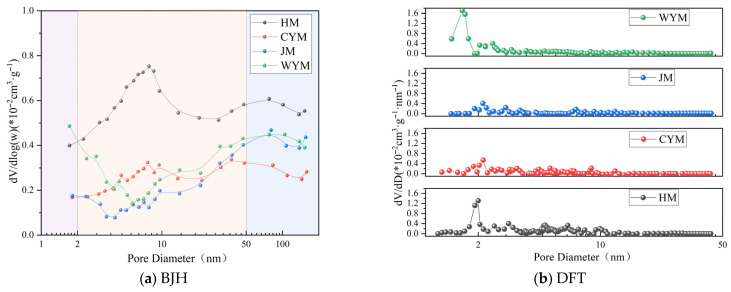
Pore distribution in coal.

Pore distribution in coal is shown in Figure 10. Pore-size distribution curves in the 2–200 nm range (BJH) show pronounced rank differentiation. With increasing coalification, the main peak position shifts toward larger pore sizes while the distribution width narrows, implying a transition from multilevel pore systems toward finer and more regular pore structures. HM exhibits a clear multimodal pattern, indicating high diversity and hierarchical pore development, including abundant mesopores and a small fraction of macropores; the distribution intensity is highest across the pore-size range, with a broad peak in the 5–20 nm interval, suggesting well-developed mesoporosity. Such pores can be associated with voids generated during early-stage thermal evolution and condensation of aliphatic/oxygenated structures and with interstitial spaces within the organic matrix. The presence of partially connected larger pores facilitates gas transport and diffusion, potentially yielding higher mass-transfer rates and adsorption potential.

By contrast, CYM and JM display flatter curves with reduced peak intensity and more subdued, near-unimodal behavior. This is consistent with increasing aromaticity and carbon skeleton densification during coalification, which tends to concentrate the pore-size distribution and can cause mesopore collapse or transformation into closed micropores. WYM shows the smoothest distribution across the full range, with pore volume contribution concentrated below 10 nm, reflecting typical high-rank pore characteristics: mesopores are markedly reduced and micropores dominate. Overall, low-rank coal exhibits a porous and open pore network, whereas high-rank coal evolves toward a micropore-dominated and comparatively closed pore system—a structural basis for the decreasing tendency of spontaneous combustion with increasing rank.

Micropore distributions in the 1–50 nm range (DFT) further highlight rank-dependent differences. WYM shows a sharp peak in the 1–2 nm interval, far exceeding the other samples, confirming that micropores dominate in anthracite. This behavior is closely linked to higher degrees of ordering/graphitization, resulting in a highly refined and more closed pore network with a narrow pore-size spread. HM exhibits a broader peak in the 2–10 nm interval with a tail extending into the mesopore region, indicating the persistence of transitional pores; these features may relate to abundant polar groups (carboxyl, hydroxyl, ether) and a higher surface energy that benefits adsorption. The peaks of CYM and JM weaken and the curves become smoother, suggesting an overall reduction in pore volume alongside a relative increase in micropore proportion and a tendency toward shrinkage and homogenization.

**Figure 11:**
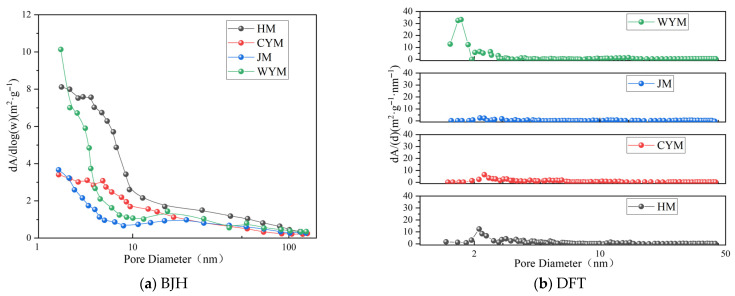
Specific surface area distribution of coal.

The specific surface area distribution of coal is shown in Figure 11. Specific surface area distributions show similar trends. For all samples, BJH surface area decreases with increasing pore size, consistent with the pore-size distribution behavior. The BJH surface areas follow HM > WYM > CYM > JM. Micropore and mesopore surface areas range from 4.2633 to 15.7096 m^2^·g^−1^ and from 1.5996 to 4.6880 m^2^·g^−1^, respectively, and both exhibit a U-shaped evolution with rank. Compared with other coals, HM shows the largest surface area at each pore-scale stage. DFT-derived total surface area is slightly higher than BJH, mainly due to the additional contribution of pores < 2 nm. With increasing rank, DFT curves evolve from multimodal to unimodal, reflecting a homogenization of pore sizes. This can be attributed to progressive dehydrogenation/aromatization and carbon-network compaction during coalification, which drive pore collapse, closure, and reconstruction and eventually produce a dense pore network dominated by micropores. 

## 3. Discussion

The results of this study indicate that coalification involves both chemical transformation of the carbon matrix and physical reorganization of the pore structure. Spectroscopic evidence consistently demonstrates that, with increasing coal rank, the degree of aromatic condensation increases while the content of heteroatoms gradually decreases. Meanwhile, adsorption measurements reveal a structural transition of the pore system from mesopore-dominated structures in low-rank coals to micropore-dominated systems in high-rank coals. Understanding this coupled evolution between macromolecular structure and pore architecture provides valuable insights into the geological evolution of coal reservoirs. Moreover, these findings have important implications for coalbed methane exploration and utilization, as well as for the injection of inert gases to suppress coal spontaneous combustion.

Future research should focus on integrating molecular-level structural characterization with advanced imaging and simulation techniques in order to further elucidate the relationship between coal macromolecular structure and pore network development. In particular, the combination of high-resolution microscopy, molecular dynamics simulations, and multiscale pore modeling is expected to provide a more comprehensive understanding of the structural heterogeneity of coal and its influence on gas adsorption and transport behavior.

## 4. Materials and Methods

### 4.1. Selection and Preparation of Coal Samples

Four coal samples were collected from the following mines: lignite from Lingquan Mine (Inner Mongolia), long-flame coal from Changtan Mine (Huineng), 1/3 coking coal from Yixin Mine (Heilongjiang), and anthracite from Baoquan Mine (Jixi). The samples are hereafter denoted as HM (lignite), CYM (long-flame coal), JM (coking coal), and WYM (anthracite). After field sampling, each coal was sealed, crushed, sieved, and vacuum-dried. Analytical samples were prepared to a particle size of 200 mesh.

### 4.2. Elemental Analysis and Proximate Analysis

The main elements of coal were determined using an Elementar Unicube elemental analyzer (Elementar, Langenselbold, Germany). Proximate analysis of the coal samples was carried out to determine moisture content (Mad), ash yield (Aad), volatile matter (Vad), and fixed carbon (FCad). Approximately 1.0 g of coal sample was weighed into a pre-dried crucible for moisture determination and dried at 110 °C for 1 h, or until a constant mass was achieved. After cooling in a desiccator, the sample was weighed and the moisture content was calculated.

For ash determination, the dried coal samples were heated in a muffle furnace, and the temperature was gradually increased to 810 °C, followed by constant heating until the mass remained stable. Volatile matter was determined by placing about 1.0 g of coal sample in a covered crucible and heating it at 900 °C for 7 min in a high-temperature furnace. Fixed carbon content was obtained by difference using the relation:

*FC* = 100 −*M_ad_* −*A_ad_* −*V_ad_* (5)

Ultimate analysis was performed using an elemental analyzer. The coal samples were completely combusted at 950–1050 °C in an oxygen atmosphere. The generated gases were subsequently reduced, and nitrogen oxides were converted to N_2_ in a reduction column. The resulting gases were separated chromatographically and quantified using a thermal conductivity detector to obtain the C, H, N, and S contents. Standard substances such as benzoic acid or acetanilide were used for calibration, and blank corrections were applied. Oxygen content was calculated by difference according to:

O = 100 − C −H − N − S − Ash − Moisture(6)

Each sample was analyzed in duplicate to ensure reliability of the results.

### 4.3. FTIR Experiment

FTIR experimental instrument was analyzed using Nicolet iS 20 (Thermo Fisher Scientific, Waltham, MA, USA) infrared analyzer. For the KBr method, 1.0 mg of coal powder was mixed with about 100 mg of spectroscopic-grade KBr, and the mixture was finely ground to ensure uniform dispersion. The mixture was then compressed into a transparent pellet under a pressure of 15 Mpa. The blended powder was then compressed into a transparent pellet using a hydraulic press under vacuum. Before sample measurement, a background spectrum was collected using a blank KBr pellet. FTIR spectra of the coal samples were subsequently recorded over the range of 4000–400 cm^−1^ with a resolution of 4 cm^−1^ and 64 scans. Finally, baseline correction, background subtraction, and normalization were performed to facilitate the identification and comparative analysis of functional groups in coal. All data processing was performed using Origin 2024b software.

### 4.4. XPS Experiment

XPS experimental instrument was analyzed using KalphaK (Thermo Fisher Scientific, Waltham, MA, USA) X-ray photoelectron spectrometer. The chemical states of heteroatoms in the coal samples were analyzed using XPS. The powdered coal samples were pressed into pellets or evenly spread onto indium foil sample holders and introduced into an ultra-high vacuum chamber with a pressure of approximately 10^−9^ mbar prior to analysis.

A monochromatic Al Kα X-ray source (1486.6 eV) was employed for excitation. Survey spectra were first recorded to determine the elemental composition of the sample surface. Subsequently, high-resolution spectra of specific regions, such as N 1s and S 2p, were collected for detailed chemical state analysis. The binding energies were calibrated using the C 1s peak at 284.8 eV as a reference. The obtained spectra were further processed by background subtraction and peak fitting to identify different nitrogen and sulfur species present in the coal samples.

### 4.5. ^13^C NMR Experiment

The experimental instrument was analyzed using Bruker Avance Neo 400WB (Bruker, Bremen, Germany) nuclear magnetic resonance analyzer. The carbon structural characteristics of the coal samples were investigated using solid-state ^13^C nuclear magnetic resonance spectroscopy with the cross-polarization magic-angle spinning (CP/MAS) technique. Approximately 100 mg of finely ground coal powder was packed into a 4 mm zirconia rotor and tightly sealed before measurement.

During the experiment, the rotor was spun at 8–12 kHz to minimize spinning sidebands. The contact time was typically set to 1–2 ms, while the repetition delay ranged from 1 to 5 s, depending on the relaxation behavior of the sample. Chemical shifts were referenced to tetramethylsilane (TMS) at 0 ppm, either directly or indirectly using an external reference standard.

### 4.6. Low-Temperature N_2_ Adsorption

The pore structure characteristics of the coal samples were analyzed using a Micromeritics ASAP 2460 (Norcross, GA, USA) surface area and porosity analyzer. Prior to the adsorption measurements, 0.3 g of each coal sample was placed in a sample tube and degassed under vacuum at 110 °C for 12 h to remove moisture and adsorbed gases.

Nitrogen adsorption–desorption isotherms were measured at −195.8 °C (77 K) using high-purity nitrogen as the adsorbate. The Brunauer–Emmett–Teller (BET) method was applied to calculate the specific surface area within the relative pressure range of 0.05–0.30. The pore-size distribution in the mesopore region (2–50 nm) was calculated using the Barrett–Joyner–Halenda (BJH) method, while the micropore structure and mesopore structure were evaluated using the density functional theory (DFT) model. Parameters including total pore volume, pore-size distribution, average pore diameter, and specific surface area were obtained to characterize the pore structural evolution of coal with different ranks.

## 5. Conclusions

(1) Aromatization and polycondensation. Based on FTIR, ^13^C NMR, and elemental analysis, coal macromolecular structure evolves systematically with rank. Low-rank HM is enriched in hydroxyl, carboxyl, and ether groups and contains well-developed aliphatic side chains, with high CH_2_/CH_3_ and the lowest aromaticity and aromatic condensation. As coalification proceeds through CYM and JM, decarboxylation, dehydration, and side-chain cleavage reduce oxygenated structures, the aromatic C=C band strengthens, aromaticity increases, and aromatic condensation becomes more pronounced. In high-rank WYM, aliphatic structures are largely removed, oxygenated groups nearly disappear, aromatic carbon exceeds 80%, and the bridgehead carbon ratio reaches 0.32, indicating highly condensed aromatic lamellae and a tendency toward graphitization. Overall, coal structure follows a ‘deoxygenation–side-chain removal–aromatization–high condensation’ pathway, with declining polarity and enhanced π–π conjugation, leading to greater structural stability.
(2) Rank-dependent incorporation of N and S. XPS results show that low-rank coal has higher fractions of N-5 and N-X, indicating that nitrogen mainly occurs at reactive edges of five-membered heterocycles or as oxidized nitrogen, which is relatively labile. With increasing rank, the fractions of N-6 and N-Q rise; particularly in JM and WYM, graphitic nitrogen increases markedly, implying progressive incorporation of nitrogen into aromatic lamellae and participation in conjugated systems. For sulfur, low-rank coal contains more thioether/thiol and partially oxidized sulfur, whereas thiophenic sulfur increases with rank and dominates in high-rank coal. Thus, N and S evolve from reactive edge structures toward stable aromatic heterocycles, reflecting stabilization and structural embedding of heteroatoms during coalification.
(3) Evolution of pore structure. Pronounced differences exist among coal ranks. HM shows a well-developed multilevel pore system dominated by mesopores (2–50 nm) with a multimodal pore volume distribution, indicative of an open pore network. As rank increases to CYM and JM, increasing aromaticity densifies the carbon skeleton, causing partial mesopore collapse or conversion into closed micropores, reducing total pore volume and concentrating the pore-size distribution. In WYM, pore sizes concentrate in 1–2 nm micropores with unimodal DFT curves, indicating a highly refined and more closed pore network. Overall, pore development exhibits a U-shaped trend, with relatively more developed pore systems in low- and high-rank coals but comparatively compact structures at intermediate ranks, highlighting the coupling between macromolecular polycondensation and pore reconstruction.

## Figures and Tables

**Table 1:** Elemental analysis and proximate analysis of coal samples.

Sample	Proximate Analysis/Wdaf %				Elemental Analysis/%				
	M_ad_	A_ad_	V_ad_	FC_ad_	C	H	O	N	S
WYM	0.62	21.28	15.13	62.97	88.50	3.31	2.04	2.61	0.4
JM	1.31	8.01	30.55	60.13	85.34	4.66	4.99	1.04	0.24
CYM	1.5	17.99	23.2	57.31	69.36	3.26	12.27	1.08	1.02
HM	1.35	20.77	39.90	37.98	67.98	4.28	20.93	0.92	0.44

**Table 2:** Structural parameters derived from ^13^C NMR of coal samples.

Sample	*f*_al_*	*f*_al_^H^	*f*_al_^O^	*f*_a_^H^	*f*_a_^B^	*f*_a_^S^	*f*_a_^P^	*f*_a_^N^	*f*_a_^C^	*f*_al_	*f*_a_	*f*_a_^’^
WYM	8.45	10.07	1.26	54.36	19.37	5.09	1.34	25.80	0	19.78	80.16	80.16
JM	15.18	4.39	4.41	42.91	16.19	15.25	1.61	33.05	0	23.98	75.96	75.96
CYM	23.49	2.94	2.43	47.03	11.69	7.15	4.12	22.96	1.07	28.86	71.06	69.99
HM	4.27	18.01	7.22	29.11	6.25	12.75	14.31	33.31	7.91	29.50	70.33	62.42

**Table 3:** Counts of different aromatic structural units in coal models.

Sample	a	b	c	d	e	f	g
WYM	1	3	3	2	2	3	0
JM	2	2	2	1	1	1	0
CYM	4	2	1	0	0	1	1
HM	6	2	0	0	1	1	0

**Table 4:** Pore volume parameters of coal samples derived from N_2_ adsorption.

Sample	BJH Total Pore Volume (10^−2^ cm·g^−1^)	BJH Pore Volume (10^−2^ cm·g^−1^)		BJH-Specific Surface Area (m^2^·g^−1^)		BET-Specific Surface Area (m^2^·g^−1^)	Average Pore Diameter (nm)
		<2 nm	2–50 nm	<2 nm	2–50 nm		
HM	1.456	0.029	0.963	0.538	0.158	7.266	9.569
CYM	0.725	0.010	0.459	0.284	2.355	3.269	11.265
JM	0.685	0.001	0.295	0.196	1.357	2.360	14.689
WYM	0.897	0.036	0.426	0.689	2.054	7.894	12.597

## Data Availability

The original contributions presented in this study are included in the article. Further inquiries can be directed to the corresponding author.

## Abbreviations

The following abbreviations are used in this manuscript:

FTIR	Fourier transform infrared spectroscopy
XPS	X-ray photoelectron spectroscopy
^13^C NMR	Solid-state 13C nuclear magnetic resonance
HM	Lignite from Lingquan mine
CYM	Long-flame coal from Changtan mine
JM	1/3 coking coal from Yixin mine
WYM	Anthracite from Baoquan mine
BET	Brunauer–Emmett–Teller method
BJH	Barrett–Joyner–Halenda method
DFT	Density functional theory
